# SerpinB2 is involved in cellular response upon UV irradiation

**DOI:** 10.1038/s41598-019-39073-w

**Published:** 2019-02-26

**Authors:** Hajnalka Majoros, Zsuzsanna Ujfaludi, Barbara Nikolett Borsos, Viktória Vivien Hudacsek, Zita Nagy, Frederic Coin, Krisztina Buzas, Ilona Kovács, Tamás Bíró, Imre Miklós Boros, Tibor Pankotai

**Affiliations:** 1 Department of Biochemistry and Molecular Biology, University of Szeged, Faculty of Science and Informatics, Szeged, Hungary; 2 Institute of Biochemistry, Biological Research Centre, Szeged, Hungary; 3 Department of Functional Genomics and Cancer, Institute of Genetics and Molecular and Cellular Biology (IGBMC), Illkirch, France; 4 Department of Pathology of the Gyula Kenézy University Hospital, University of Debrecen, Debrecen, Hungary; 5 Department of Immunology, Faculty of Medicine, University of Debrecen, Debrecen, Hungary; 6 Hungarian Center of Excellence for Molecular Medicine, Szeged, Hungary

## Abstract

Ultraviolet light induced pyrimidine dimer is a helix distortion DNA damage type, which recruits repair complexes. However, proteins of these complexes that take part in both DNA damage recognition and repair have been well-described, the regulation of the downstream steps of nucleotide excision repair (NER) have not been clearly clarified yet. In a high-throughput screen, we identified SerpinB2 (SPB2) as one of the most dramatically upregulated gene in keratinocytes following UV irradiation. We found that both the mRNA and the protein levels of SPB2 were increased upon UV irradiation in various cell lines. Additionally, UV damage induced translocation of SPB2 from the cytoplasm to the nucleus as well as the damage induced foci formation of it. Here we show that SPB2 co-localizes with XPB involved in the NER pathway at UV-induced repair foci. Finally, we demonstrated that UV irradiation promoted the association of SPB2 with ubiquitylated proteins. In basal cell carcinoma tumour cells, we identified changes in the subcellular localization of SPB2. Based on our results, we conclude that SPB2 protein has a novel role in UV-induced NER pathway, since it regulates the removal of the repair complex from the damaged site leading to cancerous malformation.

## Introduction

Our genome is constantly exposed to endogenous and exogenous sources leading to DNA damage and impairment of genome integrity. For instance, UV irradiation is an exogenous factor, which could cause formation of dimers between adjacent pyrimidine bases in the DNA^1,2^. Nucleotide excision repair (NER) is a unique pathway for the recognition and elimination a wide range of structurally diverse DNA damages, such as cyclobutene-pyrimidine dimers (CPDs), 6-4 pyrimidine-pyrimidone photoproducts (6-4 PPs), or chemically induced bulky adducts, intrastrand crosslinks caused by drugs, such as cisplatin and reactive oxygen species (ROS) induced cyclopurines^3^. The appropriate function of NER pathway requires the coordinated cooperation of various proteins, such as the XPC-Rad23B pre-complex involved in early damage recognition, the XPB and XPD helicases or XPF and XPG endonucleases taking part in the latter phases of the pathway. However, proteins involved in NER have partially been identified, some major steps regulated by ubiquitin ligases and proteases - required for latter steps of NER - have still remained to be elucidated. It has been recently published that following UV damage, DDB-Cul4 E3 ligase ubiquitylates XPC, thereby regulating its binding to the damaged site^4,5^. Furthermore, A. A. Wani *et al*. have recently described that ubiquitin specific protease 7 (USP7) is an essential protease for the NER pathway, since it deubiquitylates XPC, thereby preventing it from 26S proteasome-dependent degradation^6^. Thus, USP7 regulates the association of the pre-incision complex to the damaged site, thereby inducing the removal of UV-induced CPDs.

In addition, protease inhibitors could also negatively regulate proteolytic processes. The serine protease inhibitor superfamily, called Serpins, consists of highly heterogenic proteins taking part in tumorigenesis, blood clotting, hormone transport, inflammation, immune function and mucous production^7^. Based on the sequence similarities, the Serpin superfamily is divided into 16 different clades (A-P) and the human Serpins are represented in the first 9 clades^8^. SerpinB is the largest human Serpin clade, which consists of 13 proteins encoded in chromosome 6p25 and 18q21 regions^8,9^. It has been reported that protein members of the SerpinB clade regulate processes related to inflammation, immune system function, mucous production, apoptosis, tumour metastasis and autoimmunity^10,11^. However, no data have been published about their regulatory role in DNA repair processes, yet. SerpinB2 (SPB2), also known as Plasminogen activator inhibitor 2 (PAI-2) was first identified from placenta and it was described as an inhibitor of the extracellular urokinase plasminogen activator (uPA) and tissue plasminogen activator (tPA)^12,13^. SPB2 has been described as an intracellular Serpin involved in signal transduction, inhibition of apoptosis, macrophage survival, monocyte and keratinocyte differentiation, cyto-progression, or immune modulation^14–22^.

In this study, we have identified SPB2 as one of the most dramatically upregulated gene by UV irradiation. Here we provide evidence that the protein level of SPB2 is increased upon UV irradiation and that it is transported from the cytoplasm to the nucleus short time after damage induction. We have also found that SPB2 forms discrete nuclear foci, where the NER complex is presumably assembled and that SPB2 co-localizes with XPB protein upon UV irradiation. Based on our results, we conclude that SPB2 protein could be a potential novel player in UV-induced NER pathway. In addition, we have also found differences in the subcellular localization of SPB2 between normal and tumorous parts of the examined human basal cell carcinoma tissues. These results further support our hypothesis that SPB2 could function as a repair protein in normal cells since the translocation of the protein to the nucleus is blocked in tumorous cells thereby interfering with its DNA repair function.

## Results

### SPB2 mRNA and protein levels are increased following UV irradiation

**Figure 1:**
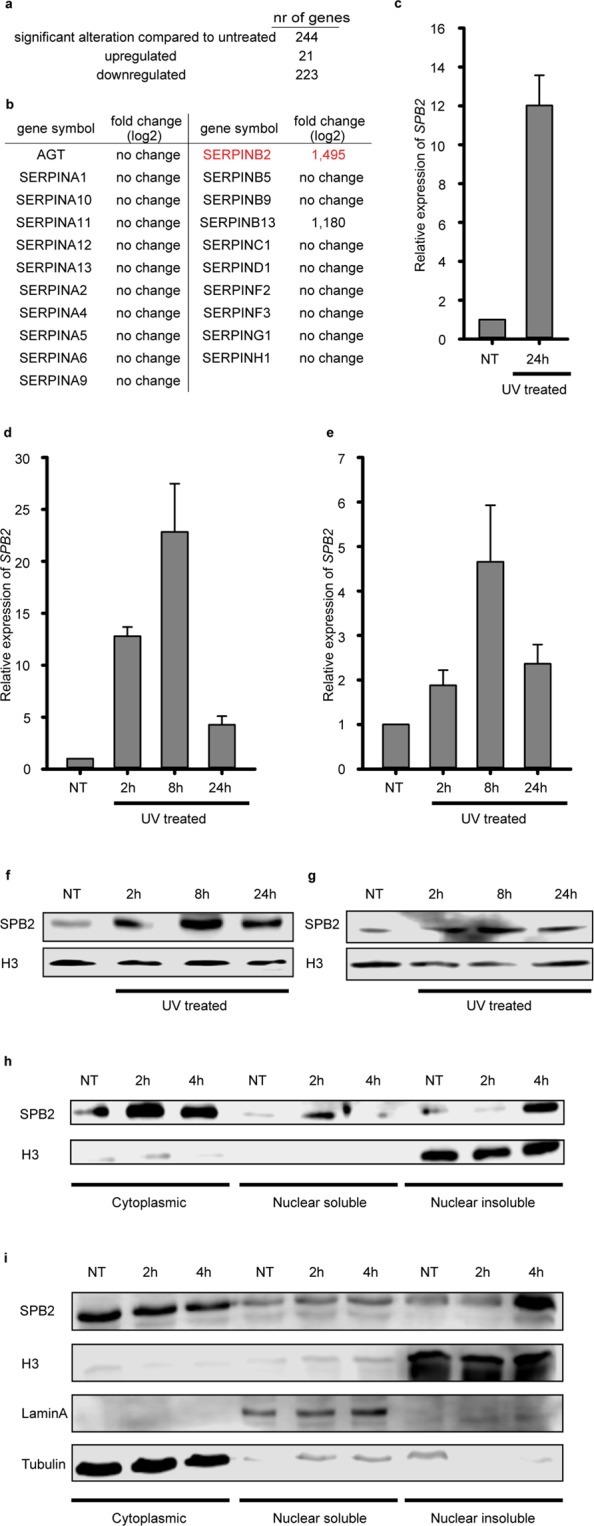
UV irradiation leads to SPB2 mRNA and protein accumulation. The means, standard deviations based on three independent experimental triplicates are indicated in case of (**c–e**). (**a**) The numbers of genes showed altered expression following UV irradiation detected by microarray experiment in Hker E6SFM keratinocyte cells. (**b**) The UV damage induced log_2_ expressional changes of the Serpin family members presented in microarray experiment are shown in Hker E6SFM keratinocytes (**c**), A375 melanoma cells (**d**) and U2OS osteosarcoma cells (**e**) by qPCR. Data were normalized to 18 S RNA. (**f,g**) Western blot detection of changes in SPB2 protein level 2-, 8- and 24 hours following UV irradiation in (**f**) Hker E6SFM and (**g**) A375 cells, respectively. H3 was used as loading control. (**h**) Western blot detection of SPB2 protein level in fractionalized lysates of U2OS cells 2- and 4 hours post-UV irradiation. H3 was used as loading control. (**i**) Western blot detection of SPB2 protein level in fractionalized lysates of A375 cells 2- and 4-hours post-UV irradiation. H3, Lamin A and Tubulin were used as loading controls.

Ultraviolet light induced DNA damage response is an extensively studied process, since improper or delayed repair of the errors could lead to cancerous malformations. In our recent study we have identified UV-induced genes in an immortalized keratinocyte cell line, Hker E6SFM, by performing a microarray experiment^23^. By comparing the mRNA profiles of non-treated and UV treated cells, 244 genes showed significantly altered expression. Twenty-one of them were upregulated and the mRNA levels of 223 genes were reduced upon UV treatment (Fig. 1a). Among the UV-induced genes, two members of the Serpin protease inhibitor family, SERPINB2 and SERPINB13, showed strong UV response, displaying 1.495- and 1.180-fold increase (log_2_), respectively (Fig. 1b). To verify the microarray data, we performed quantitative PCR (qPCR) on Hker E6SFM cells and measured the mRNA level of *SPB2* in untreated control and UV treated cells. We detected elevated *SPB2* mRNA level 24 hours following UV irradiation compared to control (Fig. 1c).

In order to analyse the expression of the *SeprinB2* gene in additional cell types, we performed qPCR experiment on melanoma cell line (A375) following UV irradiation (non-treated, 2 hours, 8 hours and 24 hours after the treatment). We observed increased mRNA level of *SPB2* 2- and 8 hours after UV irradiation and decreased mRNA level 24 hours after the treatment compared to the untreated control (Fig. 1d). To demonstrate whether UV-induced *SPB2* transcriptional activation could also be detected in non-skin derived cells, we applied U2OS osteosarcoma cell line to measure the mRNA level of *SPB2* by qPCR experiment following UV irradiation. Indeed, we could observe elevated *SPB2* mRNA level after UV irradiation compared to the control, with a similar kinetics as we detected in A375 cells (Fig. 1e vs. 1c and d).

Since our data demonstrated that UV irradiation resulted in an increased *SPB2* mRNA level, we hypothesised that SPB2 could have a role in the UV-induced cellular responses. To examine whether the increase of mRNA level could lead to the accumulation of SPB2 protein, we performed Western blot experiments on protein extracts obtained from Hker E6SFM and A375 cells following UV irradiation (Fig. 1f,g and Supplementary Fig. 1a–c). As we expected, we detected a significant increase in the protein level of SPB2 2 and 8 hours after the irradiation and it decreased 24 hours following irradiation. The protein levels showed similar pattern to that we observed in case of *SPB2* mRNA level. Besides, in Hker E6SFM cells the highest protein level was detected at 8 hours (Fig. 1f and Supplementary Fig. 1b). These results suggest that both the *SPB2* mRNA and protein levels are tightly regulated by UV irradiation.

Since we observed significant overproduction of *SPB2* 2 hours after UV irradiation, we tested whether the UV treatment influenced the subcellular localization of this protein. We performed Western blot experiment on cytoplasmic, nuclear soluble and nuclear insoluble protein fractions obtained from U2OS cells 2- and 4 hours upon UV irradiation. Under normal conditions, SPB2 is mostly localized in the cytoplasm and following UV irradiation, the protein level is increased (Fig. 1h left lanes and Supplementary Fig. 1d). Following UV-induced DNA damage, we detected increased SPB2 protein level in the nuclear soluble fraction 2 hours after the irradiation, while 4 hours after UV treatment the protein level returned to the steady-state level (Fig. 1h middle lanes and Supplementary Fig. 1d). On the contrary, we found increased SPB2 protein level only 4 hours after the UV treatment in the chromatin bound fraction of U2OS and A375 cells (Fig. 1h,i right lanes and Supplementary Fig. 1d,e). These data suggest that UV treatment induces the nuclear transport of SPB2 in a time-dependent manner 2 and 4 hours after UV treatment and activates its binding to DNA.

### Certain damaging agents targeting nucleotides could activate the SPB2 focus formation and its co-localization with XPB

**Figure 2:**
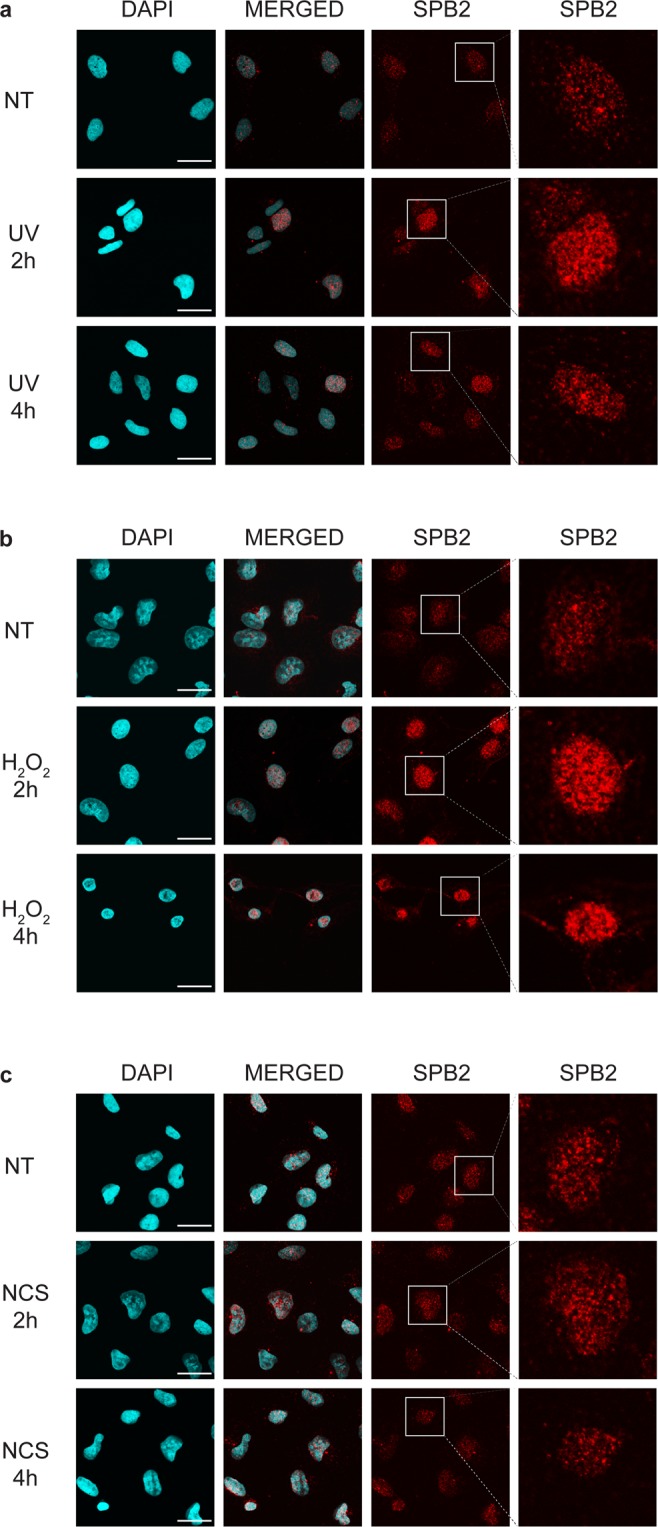
SPB2 forms nuclear foci as a response to oxidative and UV damage. Immunostaining of SPB2 (red) performed on U2OS cells upon (**a**) UV irradiation, (**b**) H_2_O_2_ and (**c**) neocarzinostatin treatment (NCS). DAPI (blue) was used to visualize the nuclei. Scale bars represent 30 µm. Only the chromatin-bound proteins were visualized. For each condition, a higher magnification of a single cell is shown on the right side of the figure. (N = 3).

To verify our previous finding that the SPB2 is transported to the nucleus upon UV irradiation and to investigate the distribution of SPB2 following DNA damage, we studied the subcellular localization of SPB2 2- and 4 hours upon UV irradiation by immunostaining. Following 2- and 4 hours UV treatments, we detected a significant higher level of SPB2 in the nucleus and we also observed that SPB2 was localized to discrete nuclear foci compared to the untreated control (Fig. 2a, Supplementary Fig. 2a). We wondered whether in addition to UV-induced DNA damage, the increased nuclear SPB2 protein level was observed upon other type of DNA damages, as well. To reveal this, we performed immunostaining on U2OS cells treated with oxidative damage inducing hydrogen-peroxide (H_2_O_2_) and DNA double-strand breaks inducing neocarzinostatin (NCS). Similar to UV damage, we found that hydrogen-peroxide treatment (2- and 4 hours) resulted in elevated SPB2 protein level in the nucleus compared to control (Fig. 2b, Supplementary Fig. 2b). On the contrary, we could not detect any significant changes in the protein level of SPB2 following NCS treatment in U2OS cells (Fig. 2c, Supplementary Fig. 2c and 3). These data showed that SPB2 protein accumulation was observed upon only those DNA damage inducing agents, which target specific nucleotides and potentially have rapid repair kinetics.

**Figure 3:**
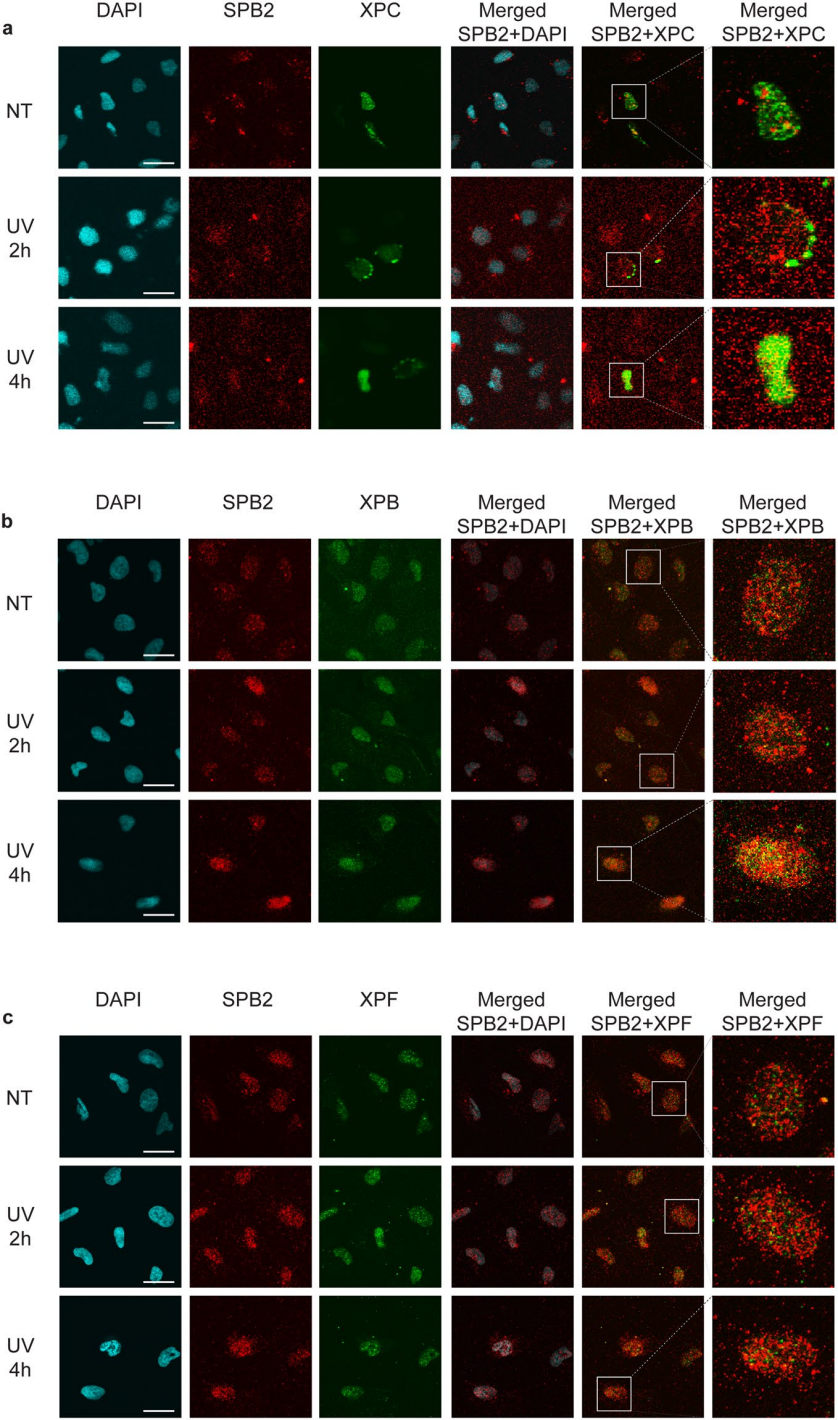
SPB2 co-localizes with XPB upon UV irradiation. (**a–c**) Co-immunostaining of SPB2 (red) and (**a**) XPC (green), (**b**) XPB (green) or (**c**) XPF (green), respectively. Only the chromatin-bound proteins were visualized by CSK-immunostaining in control (NT) and UV treated cells (UV 2 h and 4 h). Scale bars represent 30 µm. DAPI (blue) was used to visualize the nuclei (N = 3).

As UV and oxidative stress modified nucleotides are both eliminated by NER, we hypothesized that SPB2 could play a potential role in this pathway. To support this, we performed immunostaining experiment on U2OS cells to examine the subcellular localization of SPB2 and various XP proteins (Xeroderma pigmentosum) 2- and 4 hours after UV irradiation. We investigated repair proteins activated in different stages of the NER pathway, such as XPC, XPB and XPF. We found that the early factor, XPC and the late factor, XPF showed weak co-localization with SPB2 upon UV irradiation (Fig. 3a,c and Supplementary Fig. 4a,c and 5a,c). However, we observed elevated co-localization of SPB2 and XPB proteins in discrete nuclear foci 4 hours upon UV treatment (Fig. 3b and Supplementary Fig. 4b and 5b). These data suggest that SPB2 might take part in the middle steps of NER pathway probably by regulating the XPB-mediated DNA unwinding or by inactivating proteases, which are necessary for the elimination of cross-linked DNA-protein obstacles.

**Figure 4:**
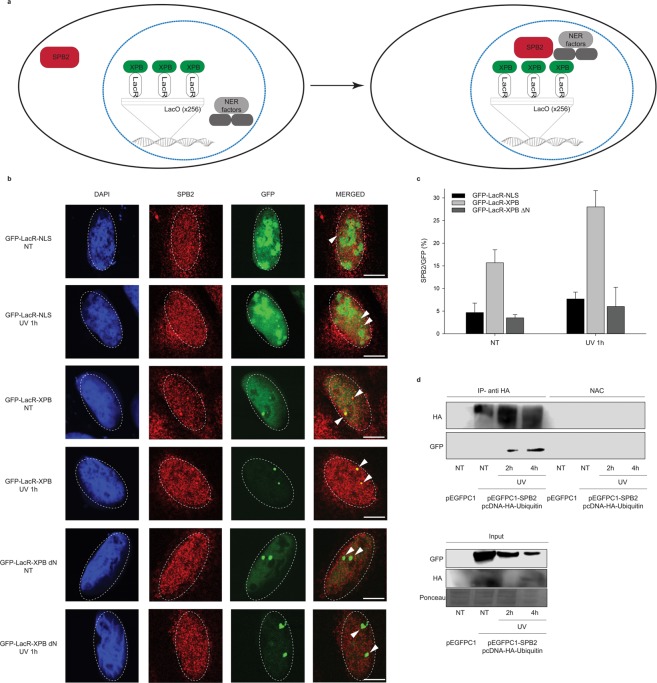
XPB could anchor SPB2 to the sites of UV damage. (**a**) Schematic representation of the LacO-tethering assay. (**b**) Recruitment of SPB2 to the anchored GFP-LacR-NLS or GFP-LacR-XPB or GFP-LacR-XPB∆N is shown in U2OS17 cells. Immunostainings were performed both under normal conditions (NT) and upon UV irradiation (UV 1 h). Arrowheads indicate the cellular position of the LacO arrays. Scale bars represent 5 µm. (**c**) The values on the graph represent the frequency of the co-localization of SPB2 with GFP obtained from 100 cells in case of each experiment (N = 3). Black columns represent the GFP-LacR-NLS, grey columns represent the GFP-LacR-XPB, while dark grey columns represent the GFP-LacR- XPB∆N data, respectively. (**d**) Immunoblot detection of SPB2 and ubiquitylated protein interaction in U2OS cells. The success of the immunoprecipitation experiment was controlled with anti-HA antibody and the connection between SPB2 and ubiquitin was detected with anti-GFP antibody (since cells were transfected with plasmid encoding SPB2-EGFP fusion protein) in the precipitated samples both in control and UV treated (2 h and 4 h) samples. Immunoblot detection of GFP and HA signals in the input samples are shown in the lower panel.

To support our data that SPB2 forms discrete repair foci with XPB, we performed LacO-tethering assay on U2OS17 cells^24^. The LacO/LacR assay consists of the LacO sequence repeated for 256 times, which could be recognized by LacR, thereby immobilizing the GFP-LacR-XPB (GFP-XPB) protein and recruiting additional NER proteins to a defined chromosomal position *in vivo*^24–26^. By this assay, we could visualize whether the SPB2 was co-localize with XPB, which allowed us to determine whether they took part in the same step of NER (Fig. 4a).

To investigate whether SPB2 is localized to XPB-LacR harboured to LacO site, we transfected U2OS17 cells with GFP-LacR-XPB vector and studied the co-localization frequency between SPB2 and fusion protein XPB compared to the GFP-LacR-NLS used as a negative control. We observed an increased frequency of co-localization between SPB2 and XPB compared to the control (Fig. 4b [lines 1 and 3] and 4c [NT samples: column 1 and 2]). To identify the protein region, which is responsible for the interaction between XPB and SPB2, we performed a LacO-tethering experiment with an N-terminally truncated, non-functional GFP-XPB fusion protein (GFP-LacR-XPB∆N) and measured the co-localization frequency of XPB∆N and SPB2. We could observe that the C-terminal part of XPB alone was unable to recruit SPB2 (Fig. 4b [line 5] and 4c [NT sample: column 3]). These results support our previous experimental data that the two proteins co-localize with each other and the N-terminal part of XPB is required for the interaction. To investigate whether DNA damages increase the interaction frequency between XPB and SPB2, we performed similar experimental setup as we described above and we also applied UV irradiation on U2OS17 cells. Upon UV irradiation, the SPB2 and XPB foci numbers were significantly increased in case of the full length GFP-LacR-XPB protein encoded vector transfection, while only low level of SPB2 could be observed both in the control (GFP-LacR-NLS) and in the non-functional GFP-XPB fusion protein expressing plasmid (GFP-LacR-XPB∆N) transfected cells (Fig. 4b [lines 2, 4 and 6] and 4c [UV 1 h samples: column 4, 5, 6]).

### UV damage regulates the ubiquitin mediated fine-tuning of SPB2

Ubiquitin signalling has been reported to participate in the regulation of various types of repair processes, such as UV-induced NER^6^. It has been already reported that upon UV irradiation, ubiquitin signal could fine-tune the function of numerous proteins involved in NER, such as XPC, which ubiquitylation is regulated by DDB-Cul4, therefore promoting the binding of XPC to the damaged DNA^4,5^. We demonstrated that XPB tethering to LacO leads to the accumulation of SPB2 at the same genomic loci. This raises the possibility that the association of SPB2 to the NER repair complex could be also fine-tuned by post translational modifications. To test whether the ubiquitylation mediated signalization is required for SPB2 loading to the damaged site, we performed co-immunoprecipitation (co-IP) experiment on U2OS cells. In this experiment to show interaction between ubiquitin and SPB2 we used GFP-tagged SPB2 and Hemagglutinin-tagged ubiquitin (HA-Ub). After co-transfection with HA-Ub and GFP- SPB2 fusion protein encoding plasmid DNA, we performed co-IP experiments on untreated and UV irradiated (2- and 4 hours) U2OS cells. In untreated cells no interaction was detected between SPB2 and Ub. On the contrary, following UV damage we found that SPB2 could be co-immunoprecipitated with HA-tagged ubiquitin both 2- and 4 hours after UV treatment (Fig. 4d, line 2). These data suggest that SPB2 may be involved in ubiquitylation mediated processes upon UV irradiation.

### SPB2 shows altered localization in basal cell carcinomas

**Figure 5:**
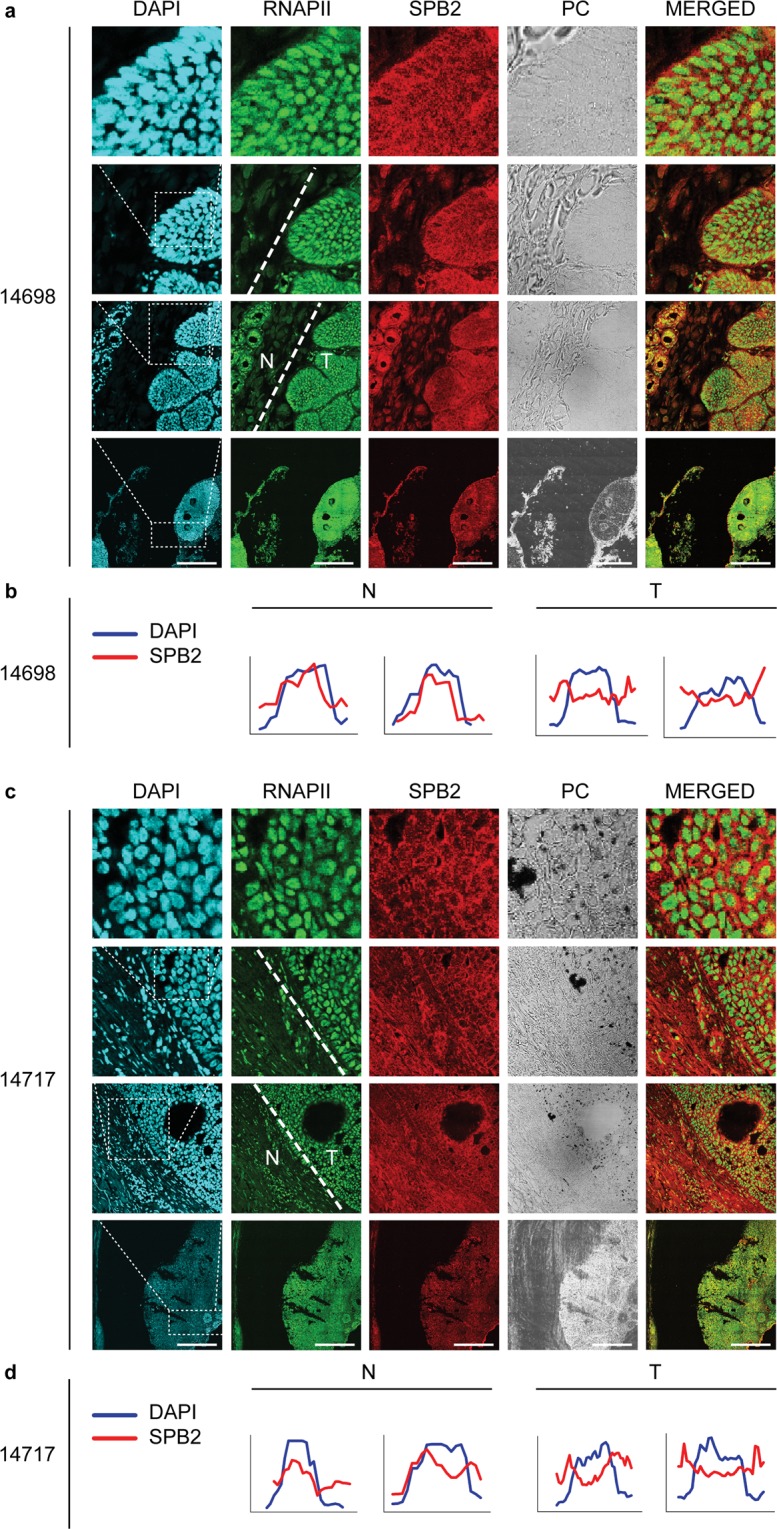
Subcellular localization of SPB2 in the normal and tumorous part of human skin tissues: (**a,c**) co-immunostaining with RNA Polymerase II (RNAPII) (green) and SPB2 (red) in normal and in basal cell carcinoma tissues. PC represents the phase contrast images. The N and T represent the normal and the tumorous part of the tissue, respectively. DAPI (blue) was used to visualize nuclei. Scale bars represent 180 µm (**b,d**) Quantitative evaluation of fluorescent intensity obtained from normal (N) and tumorous (T) part of the represented tissues. The data obtained from DAPI signal is indicated in blue, while SPB2 signal is shown in red. In each graph DAPI (blue) represents the nuclei. In the cases, when graphs of pixel intensities of DAPI and SPB2 were correlated to each other, the SPB2 showed nuclear localization (**b** normal samples). The inverse correlation of DAPI and SPB2 graphs reflects that SPB2 localizes in the cytoplasm (**d** tumorous samples).

A recent study has showed that the SPB2 is involved in cancerous malformations by affecting metastatic tumour formation. It was reported that the elevated expression of SPB2 resulted in a poor survival of bladder cancer patients^27^, or patients diagnosed with esophageal squamous cell carcinoma^28^. However, reduced expression of SPB2 also leads to reduced survival rate of patients suffering from breast^29–31^ or pancreatic cancer^32^. In this study we demonstrate that upon UV irradiation *SPB2* mRNA level is highly increased in cell types originated from skin tissue. It has been also shown that the loss of key players regulating NER leads to more frequent incidence of skin carcinoma and to poor prognosis for those patients^33,34^. Furthermore, our results demonstrate that SPB2 is an essential player in the regulation of UV-induced NER. To reveal whether SPB2 is associated with skin tumour progression, we performed immunostaining on tissue samples obtained from basal cell carcinomas. However, we could not observe any significant differences in the protein level of SPB2 by comparing the tumorous parts of the tissue with the normal parts of it. We found that SPB2 was mainly found in the cytoplasm of tumorous cells, while in the non-tumorous parts of the tissue SPB2 protein showed equal distribution between the cytoplasm and nucleus. In some cases, higher SPB2 protein level was detected in the nucleus in the normal parts of the tissues (Fig. 5 and Supplementary Fig. 6). These results suggest that SPB2 is a potential new player in UV-induced repair process. In tumorous cells SPB2 could not be transported to the nucleus and this probably leads to insufficient repair, hereby increasing the mutation rate.

## Discussion

In this study we investigated a still uncharacterized function of SPB2 protein following UV irradiation. We demonstrated that UV irradiation resulted in increased *SPB2* mRNA and protein levels in various cell types. We detected the strongest activation of *SPB2* in Hker E6SFM keratinocyte and A365 melanoma cells following UV irradiation. U2OS cells also showed similar but weaker increase in the mRNA level of *SPB2*. This could be explained by evolutionary conserved mechanisms in response to UV irradiation, which is more emphasised in keratinocyte cells, since those are normally exposed to UV light. We also demonstrated that H_2_O_2_ treatment had similar effect to UV, since it also leads to elevated SPB2 protein level. This might indicate that the activation of SPB2 upon UV irradiation is a common mechanism in different cell types, owing tissue specific repair mechanisms^35–40^, upon various DNA damages affecting nucleotides and SPB2 protein could play role in the fine-tune regulation of these repair pathways.

In addition, we also demonstrated that UV irradiation promoted the translocation of SPB2 to the nucleus and the formation of discrete nuclear foci at the sites of DNA repair. Based on these data we hypothesise that the subcellular localization of SPB2 protein is influenced by the type of DNA damage or additional stress factors. It was shown that infection or inflammation led to the cytoplasmic accumulation of the SPB2 protein^39^. However, upon UV irradiation similar cytoplasmic distribution of SPB2 was detected, the nuclear accumulation of the protein could also be observed compared to control. These results are in accord with a recent study described by Lee and Yerbury *et al*., as they showed that in a human kidney cell line SPB2-GFP fusion protein was localized in both the cytosol and nucleus^41^. Additionally, the SPB2 nuclear translocation could be tightly regulated and is necessary for performing the function of the protein shown in many cases involving transcription factors, activated MAPs, Nrf2 and also for EGFR after UV irradiation^36,42–45^. These indicate that the transport of several repair factors, and also SPB2, to the nucleus could be triggered by stress conditions affecting the integrity of the DNA, thereby promoting the process of DNA repair.

Our data suggest that SPB2 interacts with the NER complex and co-localizes with XPB protein upon UV irradiation. We also found that neither XPC, nor XPF co-localized with SPB2 upon UV irradiation. However, we did not detect physical interaction between XPB and SPB2 (data not shown), an indirect connection between them could not be excluded. Recently, it has been reported that an anchored NER factor can recruit other downstream NER factors and possible participant proteins to repair foci (but not the upstream elements of the pathway) by using LacO assay^24^. Furthermore, Salim Ziani *et al*. also showed that the formation of the preincision complex was well-regulated, since a NER factor could recruit immediate downstream factors and the recruitment of these NER elements is irreversible^24^. Finally, we showed that XPB tethering could result in the recruitment of SPB2 protein to the repair foci suggesting that SPB2 could be a potential regulator of the UV-induced cellular processes, which could be recruited to the damaged DNA region in the middle steps of the NER pathway.

NER is a well-coordinated, highly complex pathway, that has not been clearly elucidated, yet. Recent studies have reported that the ubiquitin-proteasome system (UPS) and proteases are required for the precise regulation of the NER pathway^46–48^. These studies have demonstrated that ubiquitin-mediated regulation of the NER factors is necessary only in the early steps of the repair pathway. For instance, DDB-Cul4 E3 ligase and USP7 deubiquitylase enzyme regulate the binding of XPC to the damaged DNA^4–6^. On the contrary, the regulatory effect of the USP7 in the mediator and effector steps of the NER pathway has remained unexplored, though the direct interaction between XPB and 26S proteasome has been already described^49^. Recently SPB2 has been identified as an interacting partner of the UPS system in neurons and its loss leads to disturbance in compartmentalization of the aggregated proteins, thereby resulting in malfunction of the UPS pathway^41^. Lee and Yerbury *et al*. also described that SPB2 associated with ubiquitin, although it is not ubiquitylated itself ^41^. These results are in accord with that SPB2 could be associated with ubiquitin enriched milieu. Finally, it was also reported that similar to other members of the Serpin family, SPB2 also has chaperon activity^50^. This suggests that SPB2 may act as a new regulator in the fine-tuning of the NER pathway possibly through XPB protein and the ubiquitin network.

Recently published clinical results have revealed that SPB2 has a role in gastric, breast and lung cancer^51–54^. It was also demonstrated that UV irradiation could activate the neuroendocrine system by locally induced cytokines leading to the activation of the central hypothalamic-pituitary-adrenal gland, and like this UV could regulate the central neuroendocrine system and influence the homeostasis of the body^55^. Furthermore, a strong correlation has been demonstrated between SPB2 and skin tissue originated tumorous malformations. These data also demonstrated that overexpression of SPB2 suppressed the metastasis of melanoma cells via inhibition of urokinase- (uPa) and tissue-type plasminogen activator (tPA) in extracellular space and on cell surface, thereby preventing the degradation of extracellular matrix^56,57^. However, only a few studies have revealed the connection between the intracellular level of SPB2 and tumorous malformations^58^. In this study, we did not observe any significant differences in the protein level of SPB2 in tumorous cells compared to the normal part of the tissue, but we found that SPB2 protein could be detected either in the cytoplasm or in the nucleus. In the tumorous part of the tissue, SPB2 was mainly found in the cytoplasm, while in the normal part of the tissue SPB2 protein was localized both in the cytoplasm and in the nucleus. However, in some cases we detected SPB2 protein mainly in the nucleus in the normal parts of the sections. Our data suggest that SPB2 protein may have additional function in tumour cells beside the inhibition of plasminogen activators. These results underlie the hypothesis that SPB2 promotes the repair process upon UV irradiation in the nucleus, thereby decreasing the mutation rate. Thus, in tumorous cells SPB2 could not be translocated to the nucleus to support the repair process, hereby elevating the mutation rate. Based on our results we conclude that SPB2 protein is a potential player in UV-induced NER by regulating the removal of the NER complex from the damaged DNA, and that malfunction of SPB2 could lead to tumour progression.

## Materials and Methods

### Cell lines, media and culture conditions

*U2OS17 cells* were cultured at 37 °C in DMEM (Dulbecco’s Modified Eagle Medium 4.5 g/l glucose; Lonza,) supplemented with 10% fetal bovine serum (Lonza),

U2OS osteosarcoma and A375 melanoma cells were cultured at 37 °C in DMEM (Dulbecco’s Modified Eagle Medium; Lonza) supplemented with 10% fetal bovine serum (Lonza), 4 mM L-Glutamine (Sigma-Aldrich) and 1% antibiotic (Sigma-Aldrich).

Hker E6SFM keratinocyte cells were cultured at 37 °C in Keratinocyte-SFM Medium with L-Glutamine, EGF and BPE (Thermo Fisher Scientific) supplemented with 2 mM L-Glutamine (Sigma-Aldrich) and 1% antibiotic (Sigma-Aldrich).

U2OS and A375 cell line was purchased from ATCC, U2OS17 cells were provided by Frederic Coin^24^, while Hker E6SFM cells were provided by Vilmos Tubak and were generated as described elsewhere in accordance with the relevant guidelines^59,60^. All experimental protocols were approved by the guidelines of the University of Szeged and the Medical Research Council.

### UV irradiation

This method was previously described by *Ujfaludi et al*.^23^.

Briefly, the cells were UV treated (Viber Lourmat VL-/6. LM Filtered UV lamps [Vilber Lourmat]) in a sterile chamber and UV dose of irradiation was determined by UVX Digital Ultraviolet Intensity Meter (Cole-Palmer). Cells were irradiated with half-lethal UV dose: 16 mJ/cm^2^ (U2OS) and 80 mJ/cm^2^ UV (Hker E6SFM and A375). Cells were UV treated in PBS then it was replaced by cultured media following irradiation. After that cells were incubated at 37 °C for 2, 4, 8 or 24 hours, respectively.

### H_2_O_2_ treatment

Cells were treated with 1.25 mM H_2_O_2_ and incubated for 2 hours, then the cells were washed with PBS and incubated in cultured medium for 2- and 4 hours, respectively.

### Neocarzinostatin (NCS) treatment

Cells were treated with 50 ng/ml concentration of neocarzinostatin and incubated for 15 minutes. Following the treatment, cells were washed with PBS and incubated in cultured medium for 2- and 4 hours, respectively.

### Microarray experiment

The condition used in microarray experiment and the analysis methods have been already described by Ujfaludi *et al*.^23^.

Briefly, the total RNA samples from control and UV treated Hker E6SFM cells were prepared with RNeasy Mini Kit (Qiagen) according to the manufacturer’s instructions. Each category included three independent experimental triplicates. The global expression pattern was analysed on GeneChip^®^ Human Gene 1.0 ST arrays (Affymetrix). Ambion WT Expression Kit (Life Technologies) and GeneChip WT Terminal Labeling and Control Kit (Affymetrix) were used for amplifying and labelling 250 ng of total RNA samples according to the manufacturer’s protocol. Analyses were performed by using GeneSpring^®^ GX7.3.1 (Agilent) software. Raw data (CEL files) were analysed by using the RMA algorithm. Data were normalized by using per-chip normalization (global scaling). Genes (probe sets) showed low expression rate (raw expression <100) were filtered first.

### RNA extraction, reverse transcription, qRT-PCR

Total RNA samples were isolated with ReliaPrep RNA Cell Miniprep System Kit (Promega) according to the manufacturer’s instructions. For each sample, 1 µg RNA was transcribed to cDNA by using TaqMan Reverse Transcription Reagent (Thermo Fisher Scientific). Quantitative real-time PCR reactions were performed in Thermo Scientific PicoReal Real-Time PCR System by using SYBR Green chemistry (Thermo Fisher Scientific). SPB2 specific primers 5′-GATGTGTCCACTGGCTTGGA-3′ and 5′-CCTCTCCGACATCCCTGAGA-3′ were designed by using Primer3 software. The 18 S RNA primers were used as internal control^23^. The Ct values of each sample were normalized to the internal control and the alterations in mRNA levels were calculated by the ∆∆Ct method^61^. Data were obtained from three independent experiments.

### Western blot

Western blot was performed according to a previous study^62^.

Briefly, cells were collected in lysis buffer [150 mM NaCl (Sigma-Aldrich), 1% Triton X-100 (Molar Chemicals), 50 mM Tris-HCl pH 8 (Sigma-Aldrich) and 1xPIC (Roche)] and incubated on ice for an hour, then centrifuged. The concentration of the supernatant lysates was measured with Bradford reagent (Bio Rad, 500-006) and the appropriate volumes for 30 µg of protein samples were mixed with 2xSDS loading buffer containing 5% β-mercaptoethanol (Sigma-Aldrich). Before loading, the samples were boiled for 5 minutes. The lysates were separated in SDS-PAGE, then transferred to nitrocellulose membrane (GE Healthcare). The membranes were blocked in 5% non-fat dry milk-TBST solution [20 mM Tris-HCl pH 7.4 (Sigma-Aldrich), 150 mM NaCl (Sigma- Aldrich), 0.05% Tween 20 (Molar Chemicals)]. The following first antibodies were used: anti-SPB2 (Atlas Antibodies, HPA015480) in 1:50 dilution, anti-Lamin A (Santa Cruz, sc-293162) in 1:50 dilution, anti-H3 (Abcam, ab1791) in 1:3000 dilution, anti-GFP (Abcam, ab6556) in 1:1000 dilution, anti-HA (IGBMC) in 1:2000 dilution. For chemiluminescent detection secondary antibodies were applied: RAM-HRP (Dako, P0260) and GAR-HRP (Dako, P0448) followed by incubation with Immobilon Western Chemiluminescent HRP substrate (Merck Millipore) and membranes were scanned using Li-Cor 3600 C-DiGit Blot Scanner platform.

### Fractionalized Western Blot

Cells were collected in PBS (phosphate-buffered saline) complemented with PIC (Roche), then centrifuged. The pellets were resuspended in hypotonic buffer [10 mM Tris-HCl pH 8.0 (Sigma-Aldrich), 1.5 mM MgCl_2_ (Sigma-Aldrich), 10 mM KCl (Sigma-Aldrich), 1xPIC (Roche)] and homogenized with dounce homogenizer. After cells were supplemented with sucrose buffer [20 mM Tris-HCl pH 8.0 (Sigma-Aldrich), 15 mM KCl (Sigma-Aldrich), 60 mM NaCl (Sigma-Aldrich), 0.34 mM sucrose (Sigma-Aldrich), 0.15 mM Spermine (Sigma-Aldrich), 0.5 mM Spermidine (Sigma-Aldrich), 1xPIC (Roche)], cells were centrifuged. The supernatant corresponded to the cytoplasmic extract. The pellet was resuspended in sucrose buffer. The suspension was supplemented with high salt buffer [20 mM Tris-HCl pH 8.0 (Sigma-Aldrich), 25% Glycerol (Fermentas), 1.5 mM MgCl_2_ (Sigma-Aldrich), 0.2 mM EDTA (Sigma-Aldrich), 900 mM NaCl (Sigma-Aldrich), 1xPIC (Roche)] and incubated on ice for half an hour with periodic agitation. The suspension was centrifugated. The supernatant corresponded to the soluble nuclear extract. The pellet was resuspended in sucrose buffer, then sonicated with Diagenode Bioruptor (Diagenode) for 6 times 30 sec ON, 30 sec OFF/ cycle at high power settings. The sonicated samples corresponded to the insoluble nuclear extracts, which were centrifuged. The supernatants were used for Western blot analysis.

### Cytoskeletal (CSK) Immunocytochemistry

Cells were washed with PBS (phosphate-buffered saline) then incubated with CSK buffer 3 times for 3 minutes [10 mM Hepes pH 7.0 (Sigma-Aldrich), 100 mM sucrose (Sigma-Aldrich), 3 mM MgCl_2_ (Sigma-Aldrich), 0.7% Triton X-100 (Sigma-Aldrich), 0.3 mg/ml RNase A (Roche)]. Cells were washed 3 times with PBS, then fixed with 4% formaldehyde (Sigma-Aldrich) for 10 minutes. Cells were permeabilized with 0.2% Triton X-100/PBS for 5 minutes. After washing steps, cells were blocked with 5% BSA (Sigma-Aldrich) in PBST [0.1% Tween 20 (Sigma-Aldrich) in PBS] for 20 minutes. Cells were washed with PBST, then incubated with primary antibodies diluted in 1% BSA/PBST: anti-SPB2 (Atlas Antibodies, HPA015480) in 1:300 dilution, anti-XPB, anti-XPF and anti-XPC were kindly provided by Frederic Coin. After washing steps, the following secondary antibodies were used: GAR Dylight 550 (Abcam, ab96984) in 1:600 dilution, GAM Alexa 488 (Thermo Fisher Scientific, A11029) in 1:2000 dilution. After several washing steps with PBST cells were mounted with DAPI containing ProLong Gold antifade reagent (Thermo Fisher Scientific). Samples were visualized with Olympus FluoView FV1000 confocal microscopy. In case of image capturing the same exposition-time was used for each sample. The captured images were quantified with ImageJ software.

### Immunohistochemistry of paraffinized basal cell carcinomas

Paraffinized basal cell carcinoma containing tissues, originating from the histopathological tissue bank of the Department of Pathology of the Gyula Kenézy University Hospital (University of Debrecen, Hungary), were kindly provided by Ilona Kovács; all experiments were performed and procedures were approved by the Institutional Research Ethics Committee and the Government Office for Hajdú-Bihar County (permission number DE RKEB/IKEB 4988-2018) and informed consent was obtained from all participants and their legal guardians.

Seven µm sections of paraffinized tissues were melted at 56 °C for 20 minutes. For deparaffinisation the sections were washed 3 times with xylol (Sigma- Aldrich), then washed twice with 96% ethanol and once with 70% ethanol. After that sections were permeabilized with EnVision FLEX Target Retrieval Solution Low pH buffer (Dako, K8005) for 15 minutes at temperature increasing between 65–92 °C. Then sections were blocked with 5% BSA/0.3% Triton X-100/PBS for 1 hour. To minimise the background fluorescence, the blocking buffer was supplemented with GAR HRP (Dako, P0448) and RAM HRP (Dako, P0260) in 1:2000 dilution. Samples were incubated with primary antibodies diluted in 1% BSA/PBST: anti-SPB2 (Atlas Antibodies, HPA015480) in 1:50 dilution, Pol2 7G5 (IGBMC) in 1:250 dilution. After washing steps, the following secondary antibodies were used: GAR Alexa 555 (Invitrogen, A21429) in 1:100 dilution, GAM Alexa 488 (Molecular Probes, A11029) in 1:500 dilution. Finally, cells were mounted with DAPI containing ProLong Gold antifade reagent (Life technologies). Samples were visualized with Leica SP5 confocal microscopy. The same exposition time was used for every image capturing. Microscopic pictures were analysed by ImageJ software, by using Plot profile plugin. To perform quantitative analysis first we used the microscopic figures, which represents the SPB2 localization (Red channel) and analysed the intensity of the staining along a single line. The lines were drawn as whole cell diameters, including the cytoplasm and the nucleus as well. Along this linear line we measured the fluorescence pixel intensity corresponding to SPB2 signal. The same measurement was performed for DAPI (blue), too. From the intensity data, graphs were generated, where the horizontal distance along the lines, and the pixel intensity are shown on the X- and Y-axis, respectively.

### Transfection

U2OS cells were seeded into 10 cm plates and transfected with 7.5 µg plasmid DNA using jetPEI transfection reagent (Polyplus) according to the manufacturer’s instruction. The transfection efficiency was at least 50% in each experiment. Cells were incubated for 24 hours at 37 °C and were treated with UV irradiation as described above. The applied plasmids were the followings: *pEGFPC1 empty vector, pEGFPC1-SPB2 vector, pcDNA-HA-ubiquitin vector, pEGFPC1-XPC vector*.

### Co-immunoprecipitation

This method was previously described by *Borsos et al*.^62^

Briefly, the U2OS cells were harvested in lysis buffer [150 mM NaCl (Sigma-Aldrich), 1% Triton X-100 (Molar Chemicals), 50 mM Tris-HCl pH 8.0 (Sigma-Aldrich) and 1xPIC (Roche)] and incubated on ice for an hour, then centrifugated. 250 µg protein was pre-cleared for 2 hours with 15 µl blocked Protein A-Sepharose beads (Sigma-Aldrich). In case of immunoprecipitation step, anti HA-antibody was used (IGBMC). For the detection of non-specific protein binding no antibody control (NAC) samples were included. In that case, only blocked Protein A-Sepharose beads were applied to the cell lysate. Protein-antibody complexes were collected with 40 µl blocked Protein A-Sepharose beads. After washing for four times with lysis buffer, the beads were boiled in 2xSDS loading buffer containing 5% β-mercaptoethanol (Sigma-Aldrich) for 5 minutes and centrifuged. The supernatants were used for Western blot analysis.

### LacO-tethering assay

U2OS17 cells were cultured on coverslips and transfected with 1 µg plasmid DNA using FuGENE6 transfection reagent (Promega) according to the manufacturer’s instruction. Cells were UV irradiated 23 hours after the transfection, as described above. The examined proteins were visualized by immunofluorescence staining 24 hours after transfection, as described above. Data were obtained from three independent experiments, observed 100 cells/condition. The used plasmids were the followings: *GFP-LacR-NLS vector, GFP-LacR-XPB vector, GFP-LacR-XPB ∆N vector*.

### Haematoxylin and eosin staining

Tissues were stained with haematoxylin dye, supplemented with Aluminum potassium sulfate dodecahydrate (Sigma-Aldrich). After washing with distilled water, sections were stained with 1 g/l eosin-B solution (Sigma-Aldrich). Then samples were washed twice with distilled water. The tissues were washed with isopropyl-alcohol and with distilled water, then the sections were covered. Samples were visualized with Olympus FluoView FV1000 confocal microscopy for histoarchitectural changes.

## Supplementary information

Supplementary Figure 1–6
