# Supplementary material for: SerpinB2 is involved in cellular response upon UV irradiation
Source: Sci Rep. 2019 Feb 26;9:2753. doi: 10.1038/s41598-019-39073-w (PMC6391458; doi:10.1038/s41598-019-39073-w)
Supplement: Supplementary file 1 — Supplementary Figure 1–6 [file 41598_2019_39073_MOESM1_ESM.pdf]

Supplementary Information

SerpinB2 is involved in cellular response upon UV irradiation

Hajnalka Majoros[1], Zsuzsanna Ujfaludi[1], Barbara Nikolett Borsos[1], Viktória Vivien Hudacsek[1], Zita Nagy[3], Frederic Coin[3], Krisztina Buzas [2], Ilona Kovács[4], Tamás Bíró [5,6], Imre Miklós Boros [1, 2], Tibor Pankotai[1*]

[1] Department of Biochemistry and Molecular Biology, University of Szeged, Faculty of Science and Informatics, Szeged, Hungary

[2] Institute of Biochemistry, Biological Research Centre, Szeged, Hungary

[3] Department of Functional Genomics and Cancer, Institute of Genetics and Molecular and Cellular Biology (IGBMC), Illkirch, France

[4] Department of Pathology of the Gyula Kenézy University Hospital, University of Debrecen, Debrecen, Hungary

[5] Department of Immunology, Faculty of Medicine, University of Debrecen, Debrecen, Hungary

[6] Hungarian Center of Excellence for Molecular Medicine, Szeged, Hungary

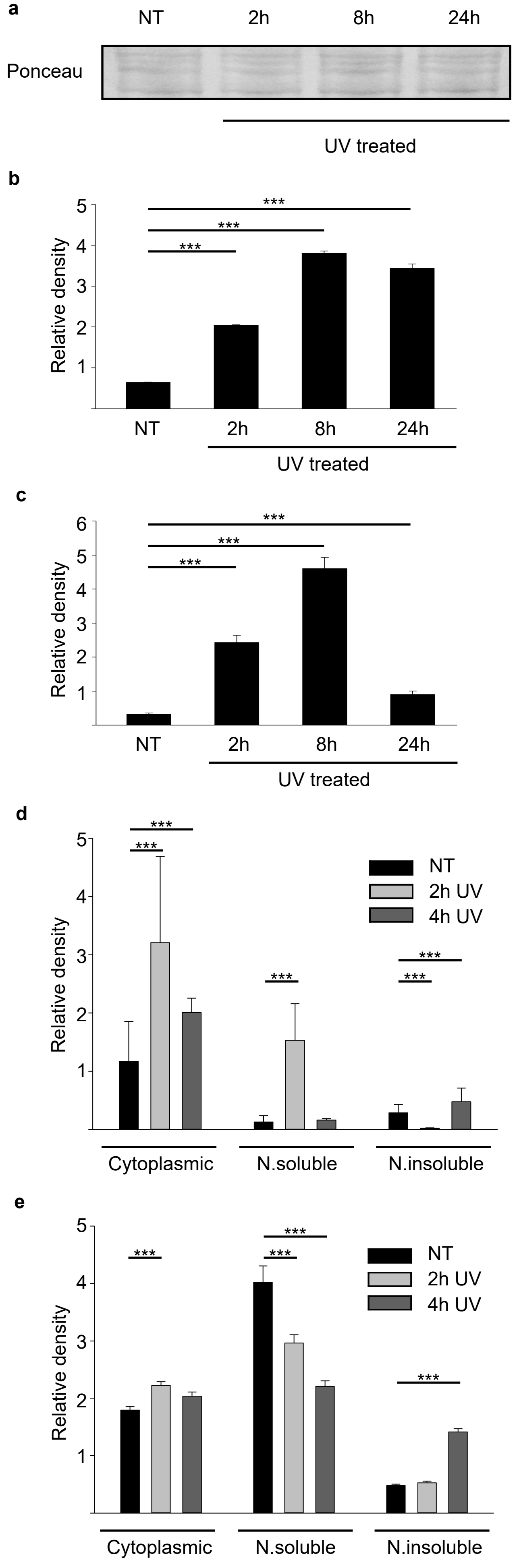

Suppl.fig.1: a) Ponceau staining was applied to show the equal loading of each sample in Western blot experiments for A375 cells. b-e) Quantification of the immunoblot experiments using Fiji (Image J) software shown on b) Figure 1f with Hker E6SFM, c) Figure 1g for A375, d) Figure 1h for U2OS cells and e) Figure 1i for A375 cells, respectively.

a

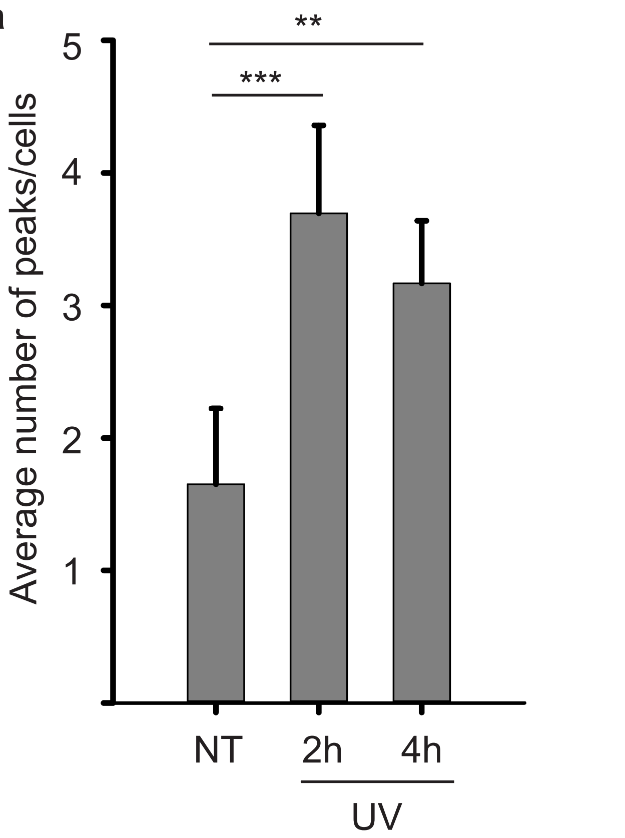

b

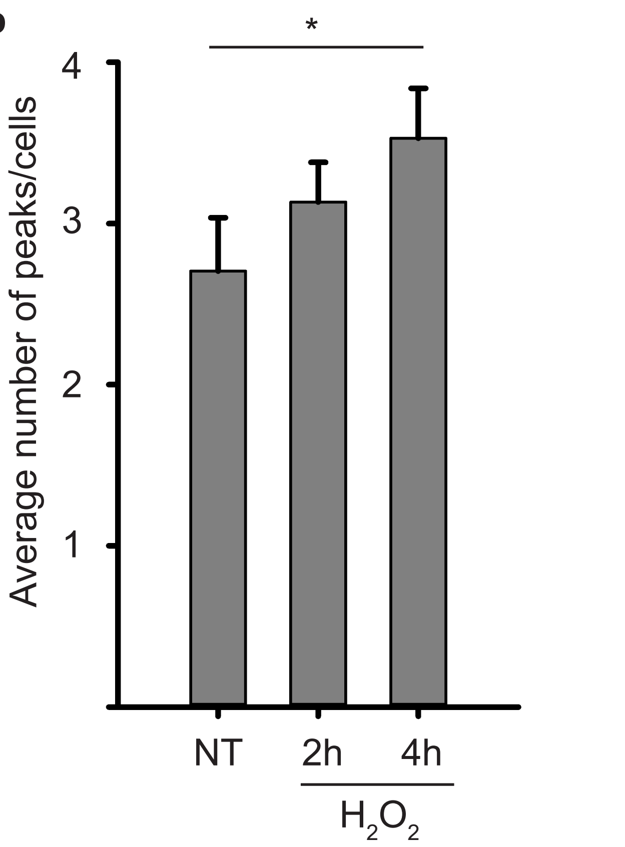

c

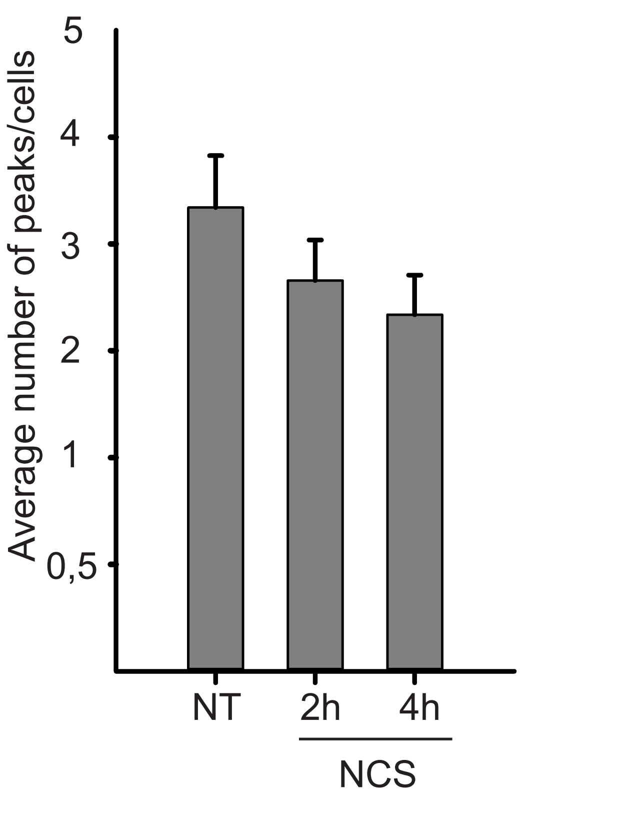

Suppl.fig.2: **ImageJ quantifications of SPB2 fluorescence intensity are rep resented on Figure2.**

Values of each graph represent the average number of peaks in a cell under normal condition (NT) and following 2 and 4 hours of (a) UV, (b) H_2O_2 and (c) NCS treatments, respectively. Significances were calculated by student t-test. Each star indicates significance between the datasets (Shapiro-Wilk, *P< 0.05, **P< 0.01, ***P< 0.001, ****P< 0.0001). The means, standard deviations and significance levels based on three independent experiments are indicated.

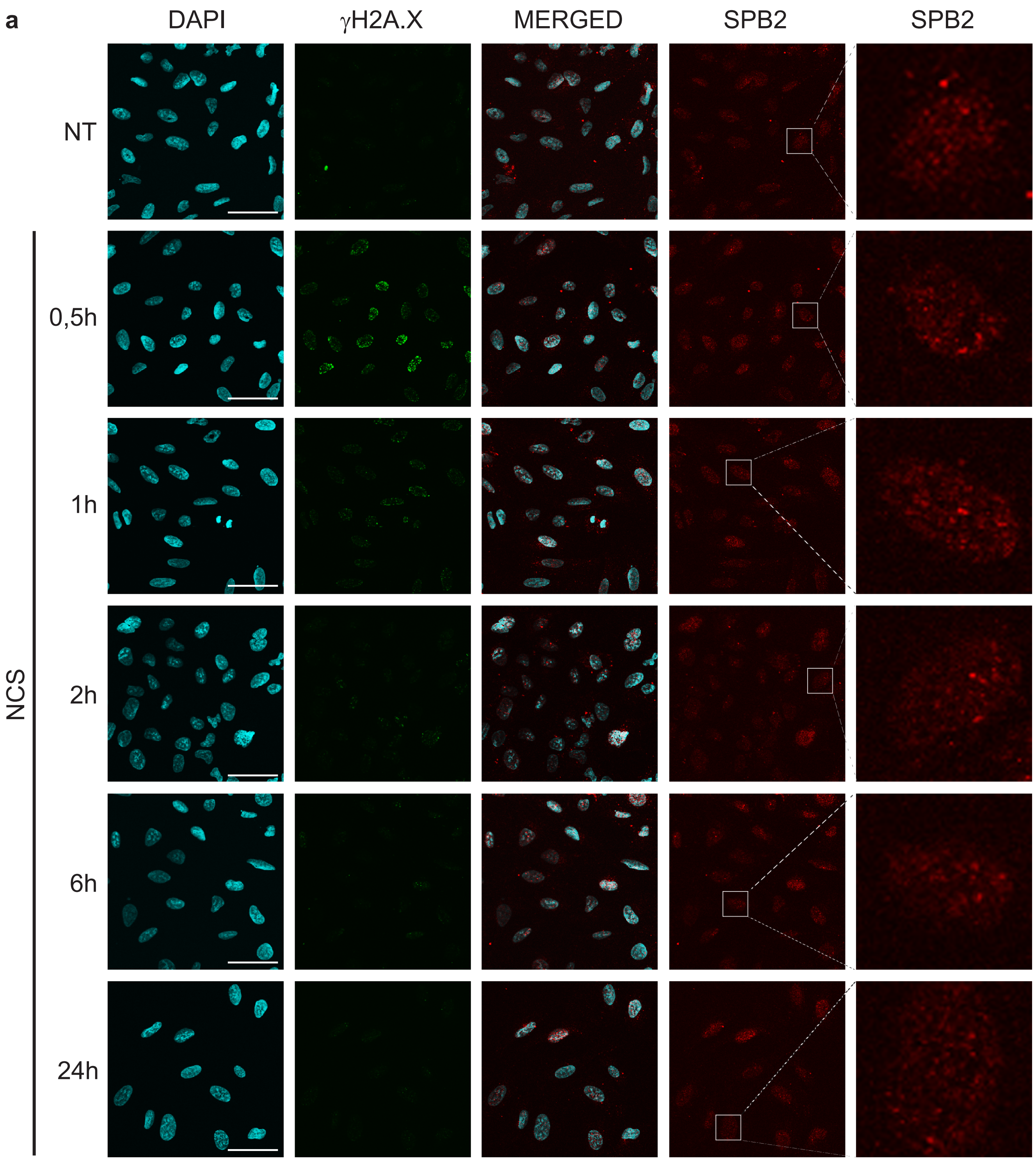

Suppl.fig.3: **SPB2 protein level does not change and form nuclear foci as a response to neocarzinostatin (NCS) treatment.** Immunostaining of SerpinB2 (SPB2) (red) upon NCS treatment is shown in U2OS cells. H2A.X S139P (γH2A.X) (green) was used to visualize the DNA double-strand breaks. DAPI (blue) was used to visualize the nuclei. The scale bar represents 30 μm. Only the chromatin-bound proteins were visualized. For each condition, a higher magnification of a single cell is shown on the right side of the figure.

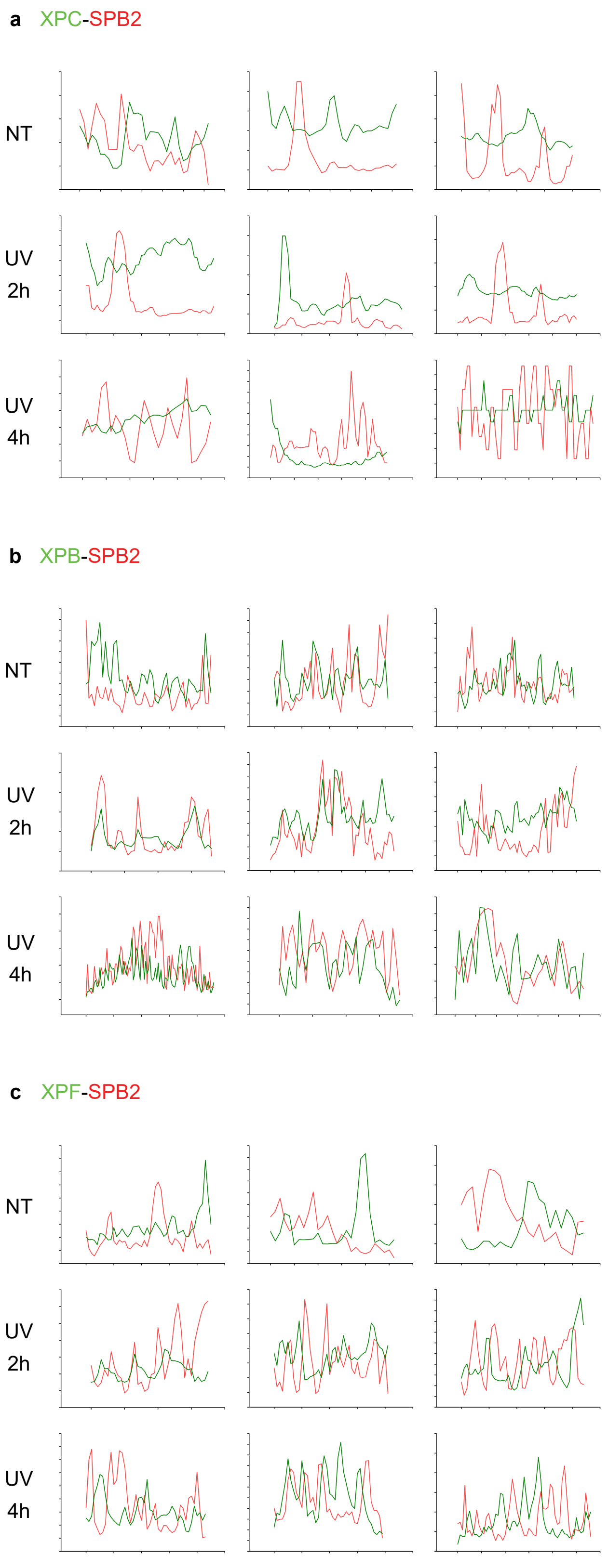

Suppl.fig.4: a-c) Quantitative evaluation of fluorescent intensity shown on Figure 3 using Fiji (Image J) software. The data obtained from (a) XPC, (b) XPB and (c) XPF signal are indicated in green, while SPB2 signal is shown in red.

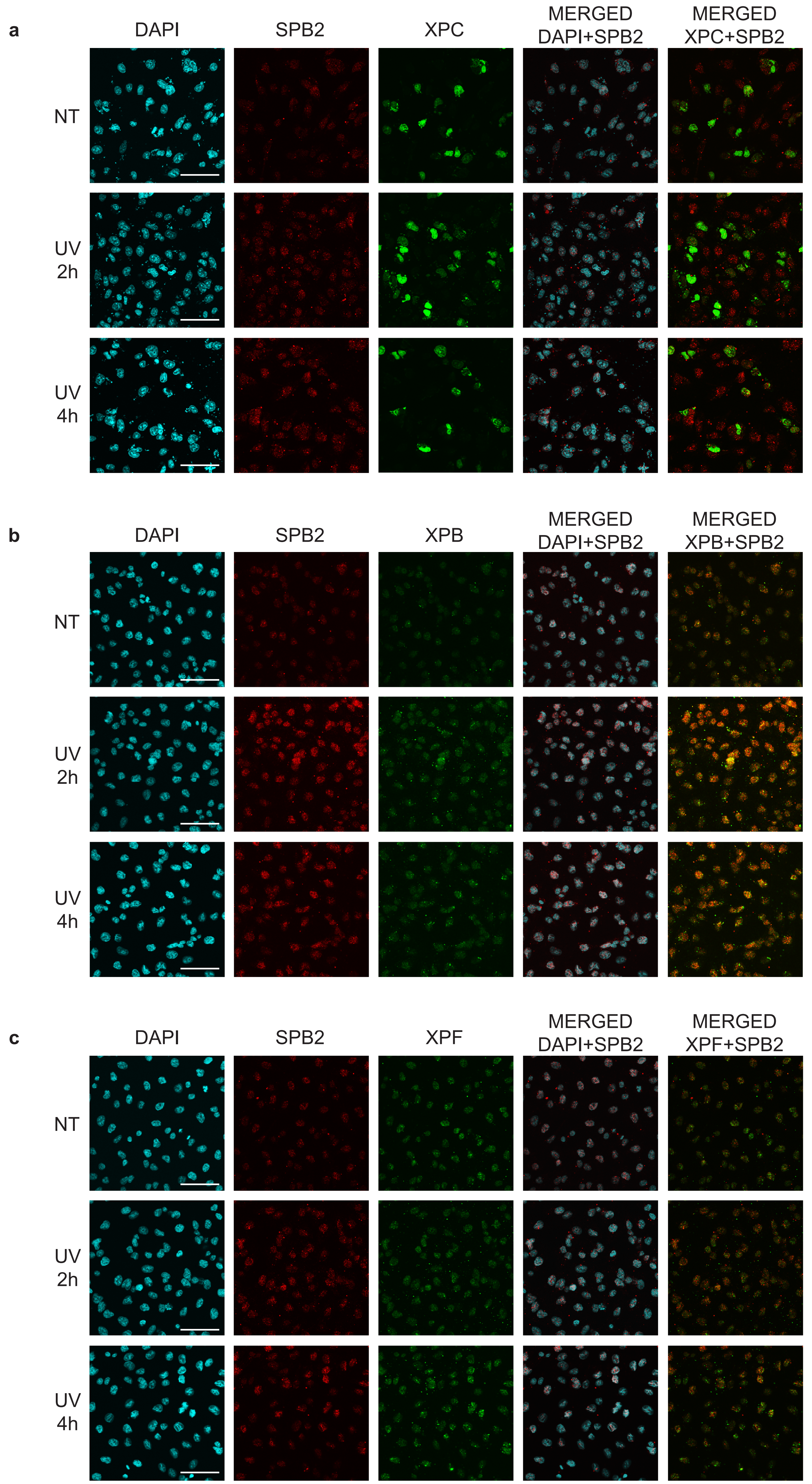

Suppl.fig.5: **SPB2 co-localizes with XPB upon UV irradiation in A375 cell line.** (a-c) Co-immunostaining of SerpinB2 (SPB2) (red) and (a) Xeroderma Pigmentosum C (XPC) (green), (b) Xeroderma Pigmentosum B (XPB) (green) or (c) Xeroderma Pigmentosum F (XPF) (green). Only the chromatin-bound proteins were visualized by CSK-immunostaining in control (NT) and UV treated cells (UV 2h and 4h). DAPI (blue) was used to visualize the nuclei. The scale bar represents 30 μm.

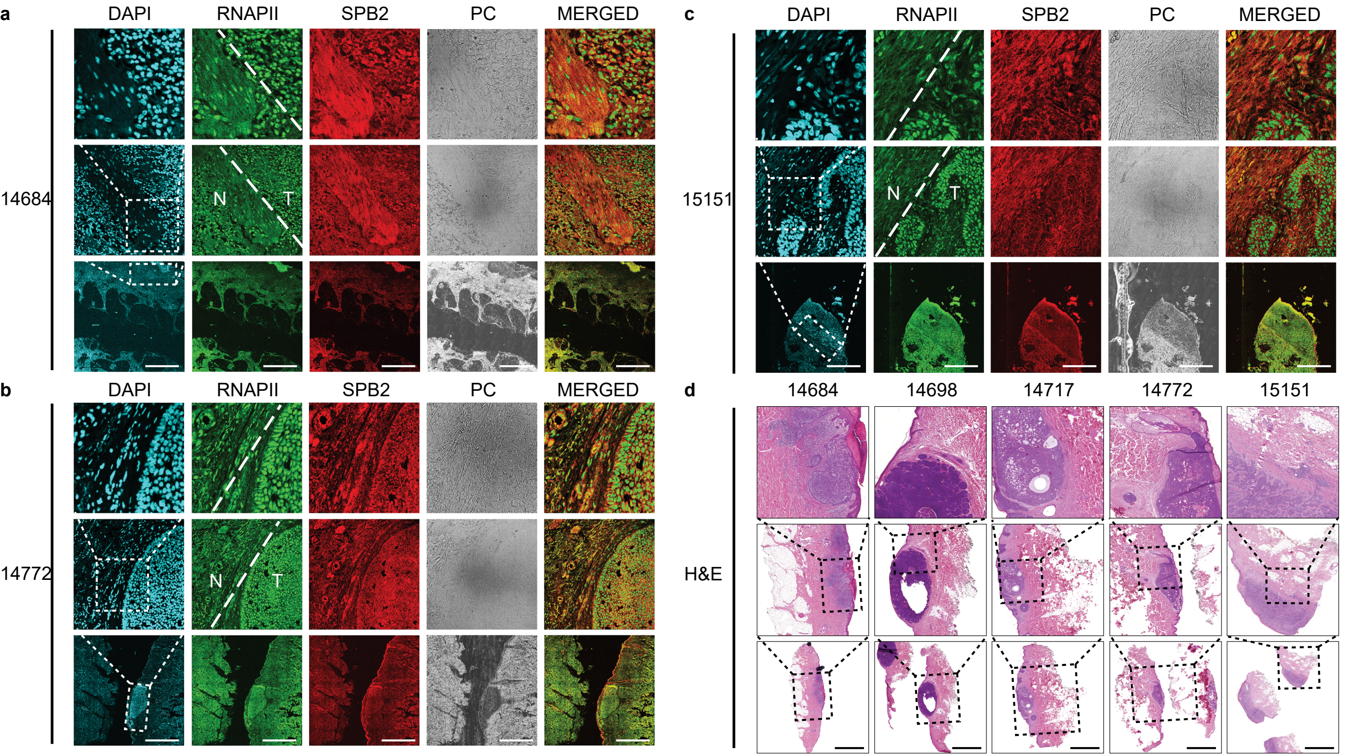

Suppl.fig 6: **Subcellular localization of SPB2 in the normal and tumorous part of human skin tissues**: (a, b and c) co-immunostaining with RNA Polymerase II (RNAPII) (green) and SerpinB2 (SPB2) (red) in normal and basal cell carcinoma tissues. PC represents the phase contrast images. N and T represent normal and tumorous part of the tissue, respectively. DAPI (blue) was used to visualize nuclei. The scale bar represents 180 μm. (d) Hematoxylin and eosin (H&E) staining of the basal cell carcinoma tissues is shown. The scale bar represents 4000 μm.
